# From clinical expert nurse to part-time clinical nursing instructor: design and evaluation of a competency-based curriculum with structured mentoring: a mixed methods study

**DOI:** 10.1186/s12912-021-00797-8

**Published:** 2022-01-04

**Authors:** Shourangiz Beiranvand, Sima Mohammad Khan Kermanshahi, Robabeh Memarian, Mohammad Almasian

**Affiliations:** 1 School of Nursing, Lorestan University of Medical Sciences, Khorramabad, Iran; 2 School of Nursing, Tarbiat Modares University, Jalal-e Al Ahmad Highway, P.O. Box 14155-4838, Tehran, Iran; 3 School of Medicine, Lorestan University of Medical Sciences, Khorramabad, Iran

**Keywords:** Competency-based curriculum, Part-time clinical nursing instructors, Clinical teaching competencies, Mixed-methods study

## Abstract

**Background:**

Transition from a clinical expert nurse to a  part time clinical nursing instructor (PTCNI) poses several challenges. Designing a professional development curriculum to facilitate the transition from a clinical expert nurse to a  PTCNI is critical to effective education. A comprehensive competency-based curriculum was developed and implemented with structured mentoring to prepare clinical expert nurses as PTCNIs.

**Methods:**

A mixed-methods study with a sequential-exploratory approach was conducted in Iran in 2019. In the qualitative phase, Saylor et al.’s (1981) seven-step model was used, consisting of (1) collecting evidence from a systematic review, (2) conducting interviews with learners, (3) setting goals and objectives, (4) design, (5) implementation, (6) evaluation, and (7) feedback. In the quantitative phase, curriculum domains were evaluated. Additionally, the effective professional communication skills module was implemented using a quasi-experimental study with a pre-test post-test single-group design for 5 PTCNIs in a pilot study.

**Results:**

After integrating the findings of the literature review and field interviews in the analysis stage, a curriculum was developed with a total of 150 h, six modules, and 24 topics. Results of the pilot study showed a significant improvement in the confidence of PTCNIs as a result of the implementation of the effective communication skills module using the mentoring method (t = − 16.554, *p* = 0.0005).

**Conclusions:**

This competency-based curriculum was based on the evidence and needs of PTCNIs and provides a complete coverage of their clinical education competencies. It is suggested that managers of educational institutes that offer nursing programs use this curriculum to prepare them in continuing education programs. Further studies are needed to thoroughly evaluate the learning outcomes for students.

## Background

Currently, the shortage of qualified nursing faculty is an international problem, as qualified nursing student applicants are turned away in countries such as Canada, China, Australia and Malaysia [1]. The most important factors contributing to this crisis are faculty aging, lack of budget, and increased job competition in clinical settings [2]. One of the innovative strategies to compensate for the shortage of nursing faculty in clinical education is the temporary employment of clinical expert nurses as PTCNIs. However, while they are experts in clinical practice, they lack the formal knowledge, skills, and attitudes to educate and evaluate students [3, 4]. According to Benner’s theory, an experienced clinical nurse is a novice educator. In other words, experience is not equal to learning or competence [5]. Successful clinical education requires skills beyond clinical expertise to best facilitate student learning. As clinical nursing education is multifaceted, clinical nursing instructors must have skills in clinical practice, effective professional communication, and skills in guiding students in applying theory in practice, evaluating students’ clinical performance, and problem solving [6]. Having expert clinicians as PTCNIs brings benefits, which include diverse clinical experience and clinical expertise, familiarity with the rules in the clinical system, having a variety of knowledge and ideas to enhance students’ learning, and cost-effectiveness over full-time faculty members. However, transfer from clinical experts to PTCNIs or adjunct nursing faculty members is challenging. Challenges reported by expert nurses include lack of formal training in clinical education skills, unawareness of methods of evaluating clinical performance and providing feedback to students, and lack of skills in establishing professional communication [7].

Providing effective clinical education brings about ongoing challenges in Iran. These include weaknesses in educational programs and content, neglect of the nursing process in clinical education, deficiencies in educational methods and evaluation of clinical performance, lack of application of theoretical education in the clinical environment, inappropriate professional interactions, and inadequate competence of clinical nursing instructors [8]. One of the important responsibilities of educational institutions is to prepare and familiarize PTCNIs for their new role as educators in clinical settings [9]. The literature has suggested orientation, mentoring, and needs-based professional development programs to prepare PTCNIs and to keep experienced faculty members up-to-date with new approaches to nursing education [7, 10]. Although many educational institutions offer orientation programs, they are often not based on logical evidence or frameworks that support clinical content, but are designed based on the hypothetical learning needs of novice instructors. Professional development programs for clinical instructors, if structured and based on competency, lead to positive outcomes, increase job satisfaction, reduce the rate of attrition, and improve the performance of clinical nursing instructors [6]. The need for a sufficient number of PTCNIs is increasing. What is unclear, however, is what competencies and professional development programs these clinical instructors need to prepare and work effectively with nursing students in clinical groups. A purposeful literature review was conducted to determine what is known about this issue.

### Literature review (preparing clinical expert nurses for the role of nursing instructors)

The first step in developing a competency-based program for nursing instructors is to identify the competencies and qualifications necessary to successfully assume their new role. Using the systematic rapid evidence assessment approach, Jetha et al.(2016) reviewed the scientific literature to identify professional development needs of novice clinical teachers to assist the transfer of clinical expert nurses to educational roles. Their findings revealed three main professional development needs for novice clinical teachers including socialization, professional development programs, and self-reflection. Based on these findings, recommendations were presented for best practices to support and prepare novice clinical teachers [11]. The available literature reports the needs of PTCNIs during role transfer as role clarification, support, clinical evaluation, teaching preparations, and orientation programs [10, 12, 13].

Various models for orienting and preparing clinical expert nurses as clinical instructors are reported in the literature. Most have emphasized the importance of faculty member development, orientation, and mentoring programs to help develop a new role and retain the role [14, 15]. Seekoe (2014) described and developed a competency-based mentoring model and critical learning theories to transfer the role of novice instructors from clinical to academic settings in South Africa. The conceptualization framework for this model included “context” (practical nurse training setting), “content” (study of mentoring resources), and “process” (mentoring needs in nursing education institutions). The mentoring takes place during the “process” through a range of activities such as relationship building, development, engagement, reflection, and assessment [16]. These models are used as a framework for developing programs to transfer clinical expert nurses from clinical practice to novice nursing instructors.

Faculty members from three nursing schools on The Eastern Shore Faculty Academy and Mentorship Initiative in Maryland, United States, have designed a program to prepare experienced nurses for new roles as PTCNIs. This 30-h program was a combination of face-to-face, simulation, online, and group mentoring sessions. The content of the program included an overview of clinical education, how to create a positive learning environment, and how to manage the multiple roles of nursing instructors. The program was implemented through mentoring and the outcome of the program included the hiring of clinical nurses as PTCNIs [17].

Wu et al. (2020). designed a web-based program for nursing preceptors in Singapore. A three-step process was applied to integrate the theoretical framework, collect evidence from a systematic review, and perform content validation by experts. The content of the program was based on a review of the literature, with results including facilitating student learning, creating a positive learning environment, evaluating clinical performance, effective feedback skills, and managing challenging situations. Part of this program was implemented as a pilot study. Nursing preceptors stated that the program content was useful for them and helped them understand the real conditions of the clinical environment [6].

Other approaches to facilitating the transition of expert nurses to novice educators is to hold workshops based on the declarations of the national league of nursing about the main competencies of nursing educators. These competencies provide a framework for identifying the basic knowledge, skills, and attitudes of educators required for curriculum design [18].

Nursing programs are challenged in employing qualified PTCNIs. They usually do not have the best approach or a suitable professional needs-based curriculum based on structured mentoring to teach PTCNIs [19, 20]. The researchers have emphasized the importance of formal and structured mentoring, which leads to increased job satisfaction, reduced role conflict and ambiguity, and increased faculty member survival [20, 21]. The aims of the study are to develop a competency-based curriculum with structured mentoring to prepare clinical expert nurses for new roles as PTCNIs and conduct pilot program evaluation.

## Methods

### Research design

**Fig. 1:**
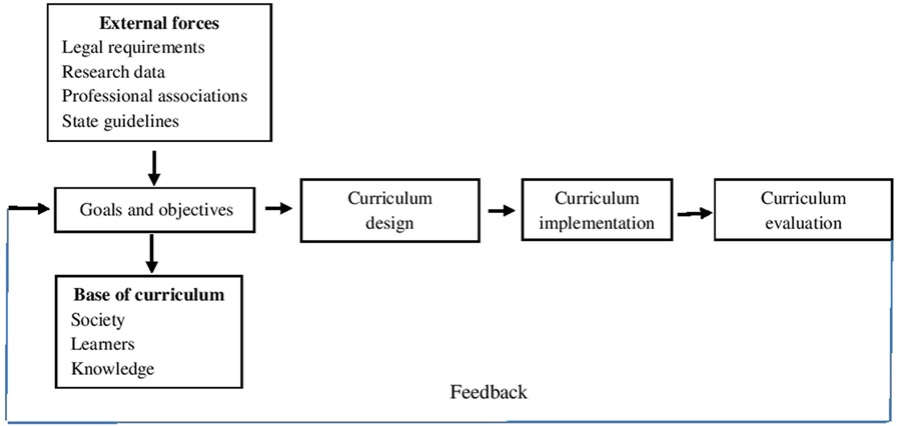
Curriculum development model of Sailor et al.(1981)

A mixed-methods sequential exploratory approach was used to design the curriculum. According to this method, the researcher explores the experiences and perceptions before conducting a quantitative study in order to gain an in-depth and comprehensive knowledge of the target phenomenon [22]. Since the ultimate goal of this study was curriculum development, the qualitative phase took precedence over the quantitative phase and the weight of the qualitative part was greater than the quantitative section. In the qualitative phase, the seven-step curriculum development model of Saylor et al. (1981) consisting of collecting evidence from a systematic review, conducting interviews with nursing instructors, setting goals and objectives, design, implementation, evaluation, and feedback was used. In the quantitative phase, curriculum domains were evaluated. Part of the curriculum using a quasi-experimental single-group pretest-posttest design as the pilot study was implemented and evaluated. In this paper, the curriculum model of Saylor et al.(1981) was chosen to design the curriculum for the following reasons:

- The application of this model is for the development and promotion of learners.
- Curriculum objectives are based on legal requirements, research data, professional associations, state guidelines, the needs of the community and learners, and knowledge [23] (Fig. 1). In order to determine what best to include in a competency-based curriculum for nursing instructors, six main steps were performed.

### Steps of curriculum design

#### Step 1: external resources, evidence from a systematic review and curriculum development

**Fig. 2:**
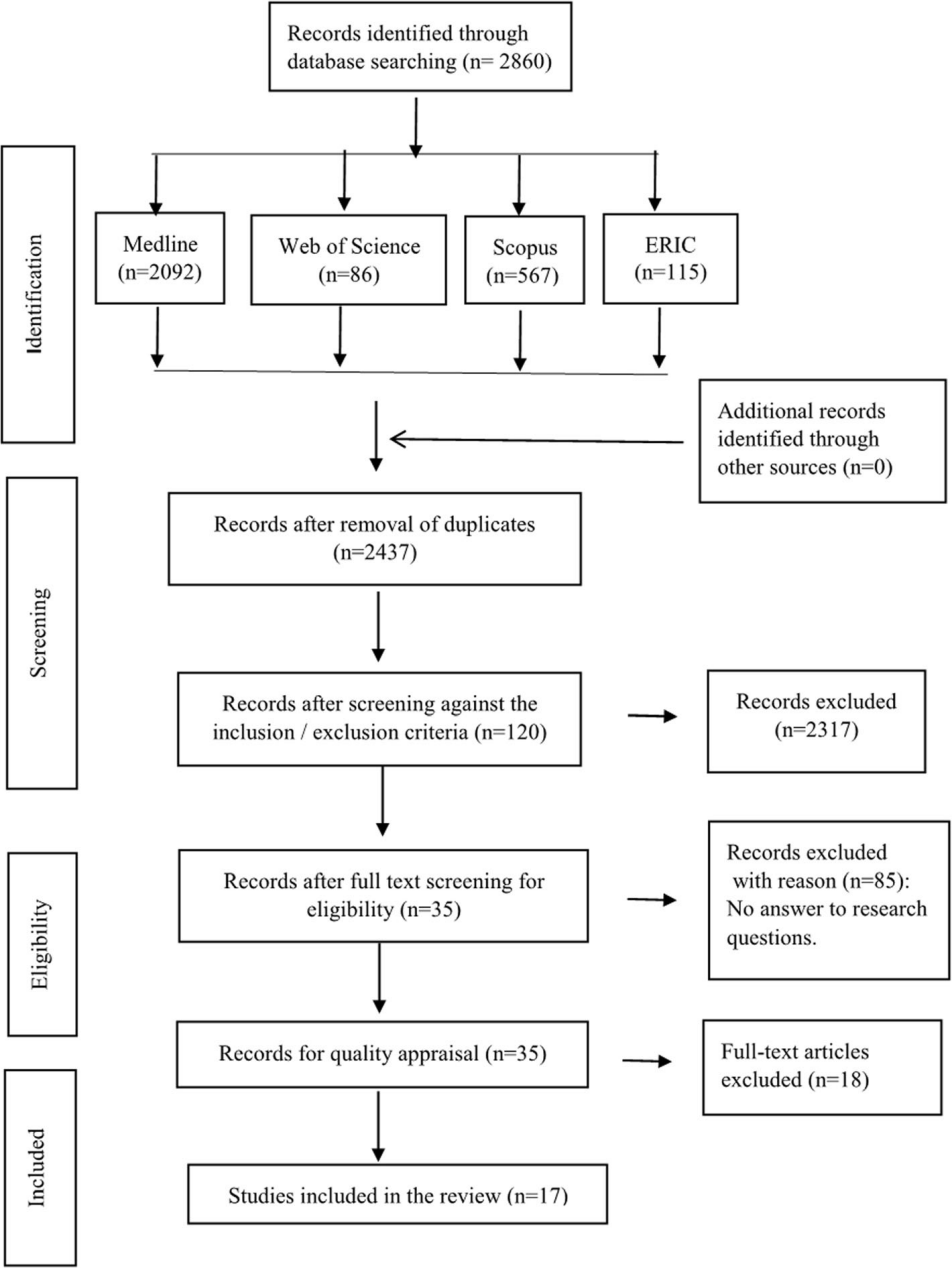
PRISMA Flow Diagram of literature search

A review was designed based on relevant criteria from the Preferred Reporting Items for Systematic Reviews and Meta-Analyses (PRISMA) checklist [24] (Fig. 2). The systematic review aimed to review and synthesize the clinical teaching competencies of PTCNIs. Four available electronic databases (Medline, Web of Science, Scopus, and Education Resources Information Centre (ERIC) were searched from 2008 to 2018. The search terms used in the search process included: (nurse OR nurses OR nursing) AND (preceptor* OR part time educator* OR novice nurse faculty OR adjunct nurse faculty) AND (competence* OR clinical skill* OR characteristic* OR qualification*). Two researchers evaluated the quality of the articles separately and in case of disagreement, the decision about the quality of the article was made through discussion. Seventeen articles were selected after quality appraisal [25].

#### Step 2: the bases of the curriculum: interviews with PTCNIs to identify their experiences, competencies, needs, and roles of the targeted learners

In this step, the research team assessed stakeholders’ needs by conducting semi-structured interviews with 15 PTCNIs from October 2018 to February 2019 in teaching hospitals in Khorramabad, Iran. The participants included nine females and six males. All ranged in age from 25 to 38 years with 5 to 15 years of clinical experience. Two PTCNIs had a master’s degree in nursing and 13 had a bachelor’s degree in nursing. The inclusion criteria were willingness to share experiences, clinical expertise in nursing, and at least 2 years of clinical education experience as PTCNIs. Based on the objectives of the study, the interview questions included the following:

1. What kind of competencies do you apply as a nursing instructor in clinical education?
2. Please describe your feelings and perceptions about your role in clinical education.

Each interview lasted 45–80 min (60 min on average). Data saturation was achieved during the last three interviews since no new concepts emerged.

#### Step 3: goals and objectives

The findings of the first and second step of the curriculum design were integrated as follows: Based on similarities and differences, the categories and subcategories of clinical education competencies of PTCNIs in the literature review and the field were compared. Duplicate categories and subcategories were removed and those that were similar were merged. Based on the results of data integration, the overall purpose of the curriculum was identified as “improving professional skills of PTCNIs” Table 1.

**Table 1:** The results of integrating the findings from the systematic review of literature and field interviews: Themes, categories, and subcategories

Themes	Categories and subcategories
1. Educational competencies	1.1. Nursing clinical instructor’s competencies in the role of teacher • Orientation of students • Having professional knowledge and practical skills • Recognizing and applying methods to facilitate students’ learning • Guiding and conducting effective group discussions 1.2. Effective professional communication • Interpersonal communication skills • Interpersonal professional communication skills
2. Supervision competencies and student support in internships	2.1. Organizing internships • Guiding and supervising students in internships • Evaluation of students’ clinical performance • Setting up a daily internship program 2.2. Supporting students • Offering emotional support • Understanding the needs and feelings of students • Motivating students • Promoting independence • Providing assistance • Giving effective feedback
3. Ethical-professional characteristics of clinical nursing instructors	3.1. Maintaining the ethical values of nursing instructors • Maintaining the dignity of students and clients • Observing ethics principles in nursing care 3.2. Professional attitude • Being a professional instructor • Being an enthusiastic instructor • Acting as a role model for professional-ethical values

#### Step 4: content of a competency-based curriculum

The modules and topics of the competency-based curriculum were determined as shown in Table 2. These were based on the results of data integration obtained from the literature review and field interviews about nursing instructors’ needs*.*

**Table 2:** Modules of the competency-based curriculum

Modules	Topics	Module description	Duration (Credit hours)
1. Effective professional communication skills	• The clinical nursing instructor’s relationship with students, colleagues, the healthcare team, and clients • Factors that facilitate and inhibit effective communication	This module emphasizes teaching, interpersonal, and professional communication skills of part-time nursing instructors.	4 h of theory and 24 h of training
2. Principles of clinical education	• Philosophy of clinical education • The strategies of preparing clinical instructors for clinical training • The strategies of preparing students on the first day of internship • Clinical education process (needs assessment, goal setting, educational planning, and evaluation) to achieve clinical outcomes • Description of clinical education models	This module explains the philosophy of clinical education, the readiness of nursing instructors and students for clinical education and the kinds of models of clinical education.	10 h of theory
3. Creative clinical education strategies	• Case method • Case study • Grand rounds • Problem solving • Critical thinking • Discussion • Clinical conference	This module provides useful and practical information about facilitating learning in clinical settings by using creative clinical education methods. Clinical nursing instructors learn how to facilitate learning in a more effective way.	4 h of theory and 24 h of skills training
4. Patient care management	• Planning patient care according to the nursing process • Principles of documentation • Principles of patient education	This module teaches the principles of client-centered and holistic care to part-time clinical instructors. It also explains the importance of patient care planning to achieve clinical teaching goals.	4 h of theory and 24 h of training
5. Supervision competencies	• Guiding and supporting students • Creating motivation and independence in students • Clinical evaluation strategies • Effective feedback techniques	This module teaches supervision competencies to clinical nursing instructors, organizing internships, providing clinical guidelines, and methods to strengthen motivation and independence in students.	4 h for theory and 24 h for skills training
6. Professional and moral characteristics of part-time nursing instructors	• Ethics in nursing care • Professionalization of students • Professional instructor	This module emphasizes the characteristics of effective clinical instructors as professional and ethical role models for students.	4 h for theory and 24 h for skills training

#### Step 5: implementation

Initially, the office of the dean of university medical science was contacted to obtain permission and to secure support and funding for the nursing instructors’ project. A budget was granted to cover the expenses of training nursing instructors. The program was implemented through structured mentoring. Instructions for mentors and mentees in this curriculum included:

- A coordinator was appointed to run the program.
- Mentors were selected from nursing faculty members with at least 5 years of educational and clinical experience, who had the ability to share their knowledge and experience with mentees and were professionally honest, motivated, and passionate.
- The matching dyads mentoring pattern was used.
- The mentees were comprehensively familiarized with the clear objectives of the mentoring program based on the selected modules.
- Workshops were developed on the topic of clinical education.
- The mentors provided timely feedback to the mentees during clinical education.

#### Step 6: evaluation

Curriculum evaluation was performed by a committee of 28 content experts consisting of 25 nursing faculty members from different higher learning institutions, two clinical nursing mentors, a hospital nurse administrator, and a representative of the nursing board in Iran.

They evaluated curriculum domains (needs, goals, content, teaching methods, assessment and evaluation, educational approach, and resources) using a checklist with the options completely stated = 3, incompletely stated = 2, and not stated = 1.

#### Step 7: feedback

In this step, the suggestions of content experts regarding the curriculum were applied and the curriculum was finalized.

### Data analysis

Data were analyzed using conventional content analysis according to the steps proposed by Graneheim and Lundman, including transcription of the data, the review of the whole interview texts for reaching a general understanding of the content, determining meaning units and initial codes, the classification of similar initial codes in broader classes, and determining the content hidden in the data [26]. At the beginning of the analysis, the full texts of the selected articles and interviews were carefully studied to achieve immersion and gain a general sense of the content. Initial codes were extracted, combined, and classified based on their similarities and through the constant comparison method. The hidden content of the data was extracted and the final codes, classes, and themes emerged with the research team’s criticism, analysis, and grouping of the codes.

### Trustworthiness

To ensure trustworthiness of the data, four criteria were used, including credibility, confirmability, dependability, and transferability [27]. The credibility of the data was approved through peer check. The research team members independently analyzed the interviews, compared the concepts, categories, and themes, and in the case of disagreement, discussed the issue to reach an agreement. To ensure the confirmability of the findings, the second and third authors, and a faculty member other than the researchers reviewed and verified the text of the interviews, along with the extracted codes and concepts. The research stages and process were recorded and reported step by step to warrant the dependability of findings. Finally, data transferability was ensured through rich and deep descriptions of the context, presenting the necessary explanations about participants’ perceptions, and using maximum variation sampling methods.

### Ethical considerations

The project was approved by the Ethics Committee of the Tarbiat Modares University, Tehran, Iran, with the code IR-TMU.REC.1396.728. At the beginning of the interviews, the objectives of the interviews were explained to the participants. They were assured that the interviews were voluntary and that their identities would not be disclosed in the research reports.

## Results

### Integrating the findings from the qualitative phase and the literature reviews

The findings obtained from the systematic review of the literature and the qualitative phase about clinical education competencies of PTCNIs were integrated in the analysis stage. Integrating the findings resulted in 20 subcategories, 6 categories, and 3 themes (Table 1).

### Content of the competency-based curriculum

The research team prepared the content of the curriculum based on the results of the interviews with the participants and the systematic review, national guidelines for clinical nursing education, and the relevant literature. The competency-based curriculum consists of six modules: 1) Effective professional communication skills, 2) Principles of clinical education, 3) Creative clinical education strategies, 4) Patient care management, 5) Supervision skills of clinical instructors, and 6) Professional and moral characteristics of clinical instructors. A brief summary of each module is presented in Table 2.

This curriculum was designed over a period of almost two years, from reviewing the literature to validation. The curriculum included workshops and clinical practice, with a total of 150 h required for teaching it (30 h for theoretical knowledge and 120 h for skills training). It will take approximately 1.5 months for each PTCNI to complete the modules. Therefore, it is expected that 4 mentors will be able to train a maximum of 12 PTCNIs in a period of 4.5 months. Upon the completion of the course, PTCNIs will receive a certificate that can be part of their professional portfolio.

### Results of curriculum evaluation by content experts

The curriculum evaluation checklist was completed by 28 content experts and emailed to the research team. Results showed that the average scores of the curriculum domains were between 2.64–2.89. The details of the mean domain ratings are shown in Table 3. Experts also commented that the duration of internships and skills training in modules should increase and the duration of theoretical courses should decrease. They also shared knowledge about the content of the curriculum. Lastly, the research team finalized the curriculum, taking into account the comments of content experts. It was presented to the Deputy Minister of Nursing of the Ministry of Health of Iran for implementation.

**Table 3:** Results of the evaluation of the competency-based curriculum domains by content experts

Curriculum domains	Not stated	Incompletely stated	Completely stated	Mean score
Needs	–	4 (14.3)	24 (85.7)	2.85
Objectives	2 (7.2)	6 (21.4)	20 (71.4)	2 .64
Educational content	1 (3.7)	5 (17.8)	22 (78.5)	2.75
Teaching methods	–	3 (10,7)	25 (89.3)	2.89
Assessment and evaluation	–	3 (10.8)	25 (89.2)	2.89
Educational approaches	1 (3.6)	4 (14.2)	23 (82.2)	2.78
References	–	3 (10.8)	25 (29.2)	2.89

### The evaluation of the curriculum modules

Mentors evaluated PTCNIs’ competencies before and after teaching each module using the nursing instructors’ job tasks scale. This instrument, developed specifically for the study, was a 9-point rating scale organized in three levels of unsatisfactory (1–3), satisfactory (4–6), and highly satisfactory (7–9). Prior to use, it was sent to 10 nursing faculty members with clinical and educational experience for evaluation and validity testing. Content validity was evaluated in terms of relevance, comprehensiveness, and appropriateness. The obtained content validity index (CVI = 0.8) indicates that the job tasks instrument has content validity.

The evaluation steps are as follows:

- Phase 1: The mentor observes and evaluates the clinical training of nursing students by PTCNIs using the job tasks instrument.
- Phase 2: The mentor teaches the required training protocols in each module to the nursing instructor in the clinical environment, the clinical instructor implements them, and the mentor gives feedback.
- Phase 3: The mentor observes and evaluates the clinical training of nursing students by PTCNIs with the job tasks instrument over four weeks after the training of each module and then provides feedback again to modify the instructor’s behavior.

### Pilot study

The main purpose of the pilot study was to evaluate the competency-based curriculum in terms of usability and quality of information. According to the review of the literature, professional communication is one of the most important competencies of clinical education of nursing instructors [28]. Effective communication between clinical instructors and students provides an ideal clinical learning environment, having a positive effect on clinical learning experiences, and increases student motivation [25]. Therefore, the module of effective professional communication skills was selected for the preliminary implementation of this program. The curriculum was implemented using a quasi-experimental single-group pretest-posttest design for 5 PTCNIs in university-affiliated hospitals in Iran in a pilot study. The professional communication competencies of nursing instructors were assessed by the observational job tasks scale before and 4 weeks after training. Then, the data were entered into the SPSS software for analysis.

### Findings related to the implementation of the professional communication skills module for PTCNIs

In this study, five expert clinical nurses were selected as PTCNIs, including 3 men and 2 women with bachelor’s degrees in nursing. The theoretical knowledge was conveyed in a 4-h lecture for nursing instructors given by a faculty member in the first week of the term. In addition, all learning goals were set out in a paper that was handed to the nursing instructors. The practical hands-on part was delivered to the instructors by means of a 24-h tutorial divided into three sessions. There was a significant difference in the mean score of the communication skills of PTCNIs before and after the intervention (Before: 1.39 ± 0.05; After: 2.74 ± 0.15, *p* = 0.0005, t = − 16.554).

## Discussion

In this study, a competency-based curriculum for PTCNIs was developed according to the 7-step curriculum development model proposed by Saylor et al. (1981) and its effects on the confidence of nursing instructors were evaluated. The competency-based curriculum is based on structural blocks of reflected competencies that move from knowledge acquisition to knowledge application [29]. The structural blocks for designing this curriculum were based on the findings of the literature review and the field interviews, including educational competencies, supervision and support competencies, and nursing instructors’ professional-ethical role modeling. Therefore, through these competencies, areas of knowledge were identified for curriculum development.

The most important components of this curriculum according to Saylor et al. ‘s (1981) curriculum model were the objectives, content, implementation, and evaluation of the curriculum. The objectives component in this curriculum was developed in the cognitive, emotional, and psychomotor dimensions. In other words, clinical instructors acquire knowledge about the competencies of clinical education, show an inclination to it, and finally acquire the necessary clinical education skills. The content, training methods, and evaluation modes were adopted according to curriculum objectives [30].

Another component of this curriculum was content development. It focuses on features such as comprehensiveness, authoritativeness, being up-to-date, and appropriateness for PTCNIs’ needs. Content development was guided by the systematic review of the literature. Therefore, this is an evidence-based curriculum. It provides a comprehensive coverage of the roles, needs, and competencies of clinical teaching for PTCNIs. Findings from other studies emphasize the importance of identifying the needs and competencies of PTCNIs via a literature review to design a program in order to prepare them for their academic roles [13, 19]. Other researchers have designed a program for nursing preceptors through a three-step process of theoretical framework, evidence from a systematic review of the literature, content validation by experts, and pretests. After the implementation of the pilot program, the preceptors stated that the content of the curriculum was useful to them and that they understood the real situation in the clinical environment [6]. Another study has also used the Delphi technique and literature review to prepare the contents of a competency-based curriculum [31].

In addition to being evidence-based, the content of this curriculum is based on assessing the needs and experiences of learner PTCNIs. The curriculum is considered dynamic when learners are involved in the learning process. If the selected content is much more challenging than the learners are able to deal with, the target concepts and skills will not be understood. However, if the content is too trivial and facile in comparison with learners’ capabilities, learners will have no desire for a positive change via learning [32]. Some researchers have designed the content of the midwifery training curriculum in Beijing based on the results of interviews with learners about their specialized training needs [33]. Therefore, identifying needs in the review of literature and field interviews were the cornerstone for the design of this curriculum.

This curriculum was implemented through the structured mentoring model, which is another component of this curriculum. Mentoring refers to the idea of encouraging learners to identify their learning needs so that they can complete their learning process based on self-regulation and self-reflection [34]. The results of a systematic review by Nowell et al. (2017) indicated that there is an obvious gap in describing the processes in mentoring programs. They reveal a lack of consistent and structured mentoring in academic settings and also indicate that nursing faculties lack evidence-based guidance about where to begin in developing and implementing mentorship programs [20].

The current curriculum was designed during a semester with specific components of mentoring, including choosing a coordinator to run the program, using the matching dyads mentoring pattern, setting specific objectives, frequent and continuous communication between the mentors and the mentees, the development of clinical teaching workshops, and providing appropriate feedback. Therefore, this study is of great importance in order to address this gap in the literature by developing a curriculum with structured mentoring and components specifically designed for PTCNIs.

The final component of this curriculum was evaluation. Experienced faculty members in various fields of nursing, including clinical nursing and nursing education, evaluated the content of this curriculum. The results of content validation showed that the content of the competency-based curriculum has a good level of validity. Qualitative feedback from content experts on the elements of the competency-based curriculum ensured that curriculum patterns and concepts are relevant.

In the quasi-experimental pilot study, significant differences were observed between the pre-intervention and post-intervention scores, which demonstrated increases in PTCNIs’ confidence regarding the implementation of the communication skills module in clinical settings. These results are consistent with several previous studies by other researchers in which a mentorship program improved PTCNIs’ competencies [17, 19].

Clinical education is imperative for developing qualified nursing students, who are prepared for professional practice. This cannot be achieved without confident clinical instructors. Academic administrators of nursing programs need professional development programs to prepare PTCNIs for clinical education of students, so that they meet the expectations of the nursing program and the nursing profession [19]. Developing a proper competency-based curriculum provides learners with the opportunity to evaluate their previous skills and knowledge, while they acquire knowledge and learning principles about clinical training competencies. Those who complete this curriculum can use their new knowledge and skills to take on the role of clinical instructors to strengthen the link between the clinical and academic environments.

Some components of this competency-based curriculum were determined based on the specific needs of PTCNIs in the study’s locality. Therefore, research findings can have contextual limitations. The seven-step process of curriculum development is time consuming. Therefore, delays may occur in the development of a competency-based curriculum. It is possible that the positive outcomes observed in the pilot study could be attributed to the small self-selected group of 5 nursing instructors selected by convenience sampling; thus, some selection bias may have affected the results and further investigations are needed. The outcomes related to nursing students’ satisfaction and competence were not evaluated. The next step could be further large-scale experimental studies to investigate the implications of this curriculum and to evaluate its effects on student’s satisfaction and clinical performance.

## Conclusion

This study used an innovative 7-step process to develop a competency-based program for PTCNIs. The unique features of this competency-based curriculum are that it is based on research data, learners’ needs, and interactive features between mentors and mentees in a structured mentoring program. This curriculum completely covers the clinical education competencies of PTCNIs in the areas of clinical teaching abilities, ethical-professional role modelling, supervision skills, and evaluation in clinical environments. Part of this competency-based curriculum for clinical instructors was conducted as a pilot study. The outcome of the pilot study was used to improve the curriculum. It is suggested that managers of educational institutions in nursing programs use structural mentoring curriculums to prepare PTCNIs in continuing education programs.

## Data Availability

The data and all supporting materials used in our manuscript are freely available to any scientist wishing to use them from the corresponding author on request.

## Abbreviations

PTCNIs: Part-Time Clinical Nursing Instructors

## References

[1] Sorrell JM, Cangelosi PR (2015). Expert clinician to novice nurse educator: Learning from first-hand narratives.

[2] American Association of Colleges of Nursing (2020). Fact sheets, nursing faculty shortage.

[3] Wenner TA, Hakim AC (2019). Role transition of clinical nurse educators employed in both clinical and faculty positions. Nurs Educ Perspect.

[4] Clochesy JM, Visovsky C, Munro CL (2019). Preparing nurses for faculty roles: the institute faculty recruitment, retention and mentoring (INFORM). Nurse Educ Today.

[5] Benner P, Sutphen M, Leonard V, Day L, Shulman LS. Educating nurses: A call for radical transformation. San Francisco, CA: Jossey-Bass; 2009. https://www.wiley.com/en-us/Educating+Nurses%3A+A+Call+for+Radical+Transformation-p-9780470457962.

[6] Wu XV, Chi Y, Chan YS, Wang W, ENK A, Zhao S (2020). A web-based clinical pedagogy program to enhance registered nurse preceptors' teaching competencies–An innovative process of development and pilot program evaluation. Nurse Educ Today.

[7] Morrison L. Assessing part-time nursing faculty needs: A needs assessment for a quality improvement project. Teach Learn Nurs. 15(1):42–4. 10.1016/j.teln.2019.08.011.

[8] Jasemi M, Whitehead B, Habibzadeh H, Zabihi RE, Rezaie SA (2018). Challenges in the clinical education of the nursing profession in Iran: a qualitative study. Nurse Educ Today.

[9] Billings DM, Halstead JA (2019). Teaching in Nursing e-Book: A guide for faculty.

[10] Summers JA (2017). Developing competencies in the novice nurse educator: an integrative review. Teach Learn Nurs.

[11] Jetha F, Boschma G, Clauson M (2016). Professional development needs of novice nursing clinical teachers: A rapid evidence assessment. Int J Nurs Educ Scholars.

[12] McPherson S (2019). Part-time clinical nursing faculty needs: an integrated review. J Nurs Educ.

[13] Owens RA (2017). Part-time nursing faculty perceptions of their learning needs during their role transition experiences. Teach Lean Nurs.

[14] Schoening AM (2013). From bedside to classroom: the nurse educator transition model. Nurs Educ Perspect.

[15] Clark CL (2013). A mixed-method study on the socialization process in clinical nursing faculty. Nurs Educ Perspect.

[16] Seekoe E (2014). A model for mentoring newly-appointed nurse educators in nursing education institutions in South Africa. Curationis.

[17] Reid TP, Hinderer KA, Jarosinski JM, Mister BJ, Seldomridge LA (2013). Expert clinician to clinical teacher: developing a faculty academy and mentoring initiative. Nurse Educ Pract.

[18] Gilbert C, Womack B (2012). Successful transition from expert nurse to novice educator? Expert educator: it's about you!. Teach Learn Nurs.

[19] McPherson S, Candela L (2019). A delphi study to understand clinical nursing faculty preparation and support needs. J Nurs Educ.

[20] Nowell L, Norris JM, Mrklas K, White DE (2017). A literature review of mentorship programs in academic nursing. J Prof Nurs.

[21] Poorman SG, Mastorovich ML (2017). Promoting faculty competence, satisfaction and retention: faculty stories supporting the crucial need for mentoring when evaluating nursing students. Teach Learn Nurs.

[22] Creswell JW, Creswell JD. Research design: Qualitative, quantitative, and mixed methods approaches: Sage publications; 2017. https://www.google.com/books/edition/_/KGNADwAAQBAJ?hl=en

[23] Saylor GJ, Alexander WM, Lewis AJ (1981). Curriculum planning for better teaching and learning.

[24] Moher D, Liberati A, Tetzlaff J, Altman DG, for the PRISMA Group (2009). Preferred Reporting Items for Systematic Reviews and Meta-Analyses: The PRISMA Statement.

[25] Beiranvand S, Kermanshahi S, Memarian R (2021). Nursing instructors’ clinical education competencies: an integrated review. J Pak Med Assoc.

[26] Graneheim UH, Lundman B (2004). Qualitative content analysis in nursing research: concepts, procedures and measures to achieve trustworthiness. Nurse Educ Today.

[27] Streubert HJ, Carpenter DR. Qualitative research in nursing: advancing the humanistic imperative. 5th ed. Philadelphia: Wolters Kluwer Health/Lippincott Williams & Wilkins;2011. https://www.amazon.com/Helen-Streubert-Speziale/e/B001IO9MT2%3Fref=dbs_a_mng_rwt_scns_share

[28] Valiee S, Moridi G, Khaledi S, Garibi F (2016). Nursing students' perspectives on clinical instructors' effective teaching strategies: a descriptive study. Nurse Educ Pract.

[29] Schumacher G, Risco K (2017). Competency-based nurse practitioner education: an overview for the preceptor. J Nurse Pract.

[30] Zeller MP, Sherbino J, Whitman L, Skeate R, Arnold DM (2016). Design and implementation of a competency-based transfusion medicine training program in Canada. Transfus Med Rev.

[31] Raghav PR, Kumar D, Bhardwaj P (2016). Experience of Delphi technique in the process of establishing consensus on core competencies. Int J Appl Basic Med Res.

[32] Andronache D, Bocoş M, Neculau BC (2015). A systemic-interactionist model to design a competency-based curriculum. Procedia Soc Behav Sci.

[33] Bo H, Zhang D (2018). Design of curriculum for specialized midwives training based on investigation of needs of midwives in Beijing. Int J Nurs Sci.

[34] Koenen AK, Dochy F, Berghmans I (2015). A phenomenographic analysis of the implementation of competence-based education in higher education. Teach Teach Educ.

